# An Update on Cardiovascular Risk Factors After Kawasaki Disease

**DOI:** 10.3389/fcvm.2021.671198

**Published:** 2021-04-16

**Authors:** Yuan-Yuan Zeng, Min Zhang, Syeun Ko, Feng Chen

**Affiliations:** ^1^Department of Pharmacy, Children's Hospital of Nanjing Medical University, Nanjing, China; ^2^Department of Pharmacy, Boston Medical Center, Boston, MA, United States; ^3^School of Pharmacy, Northeastern University, Boston, MA, United States

**Keywords:** Kawasaki disease, atherosclerosis, long-term management, cardiovascular risk factors, endothelial dysfunction, multisystem inflammatory syndrome

## Abstract

First described in Japan 50 years ago, Kawasaki disease is a worldwide multisystem disease. It is an acute self-limited vasculitis of unknown etiology that can lead to coronary artery lesions, such as dilatation, aneurysms, and stenosis in children. It is one of the common causes of acquired heart disease among children in developed countries. The coronary aneurysm is a severe complication in the acute stage, possibly leading to stenotic lesions or myocardial ischemia. More concerns have centered on endothelial damage and the early onset of atherosclerosis in patients with KD. Although the coronary artery aneurysm is small or degenerated, the vascular structure does not return to normal, vascular endothelial dysfunction and remodeling continue. Most patients diagnosed with coronary artery sequelae are at risk of long-term complications. There are still many unknown aspects regarding the long-term prognosis of patients. Concerns have centered on the early onset of atherosclerosis in patients with KD. There is still no consensus on the relationship between Kawasaki disease and atherosclerosis. This study aimed to evaluate if patients with a history of KD were at risk of accelerated atherosclerosis.

## Introduction

Kawasaki disease (KD) is a form of idiopathic vasculitis that affects small- or medium-sized vessels throughout the body, and often the coronary arteries (1). It is considered the leading cause of acquired heart disease among children living in developed countries (2). Early diagnosis and treatment of KD have reduced the incidence of coronary artery abnormalities (CAAs) from ~25 to 5% (3). Apart from CAAs, children with KD are also prone to develop endothelial dysfunction (4, 5), arterial stiffening (6, 7), and a proatherogenic lipid profile (8). Although there is sufficient data on CAAs related to KD, data on long-term complications of KD, especially atherosclerosis, remain limited. In the late-stage of KD, markers of atherosclerosis such as endothelial dysfunction, oxidative stress, elevated levels of high-sensitivity C-reactive protein (hsCRP)/C-reactive protein (CRP), and inflammatory cytokines have been reported (9–13). The accumulating data suggests that patients with KD history may be at risk of developing premature atherosclerosis. The American Heart Association (AHA) guidelines in 2004 (14) stratified patients with KD according to their relative risk of myocardial ischemia and recommend monitoring those with extensive CAA or coronary artery obstruction for known risk factors of atherosclerosis. The updated AHA 2017 guidelines (15) modified the risk stratification criteria by incorporating different risk levels based on past and current coronary artery involvement and adjusting for body size, which the previous guidelines did not address. In this study, we sought to provide updates on the profile of cardiovascular risk factors in KD patients after the acute illness.

## Epidemiology of KD

**Figure 1:**
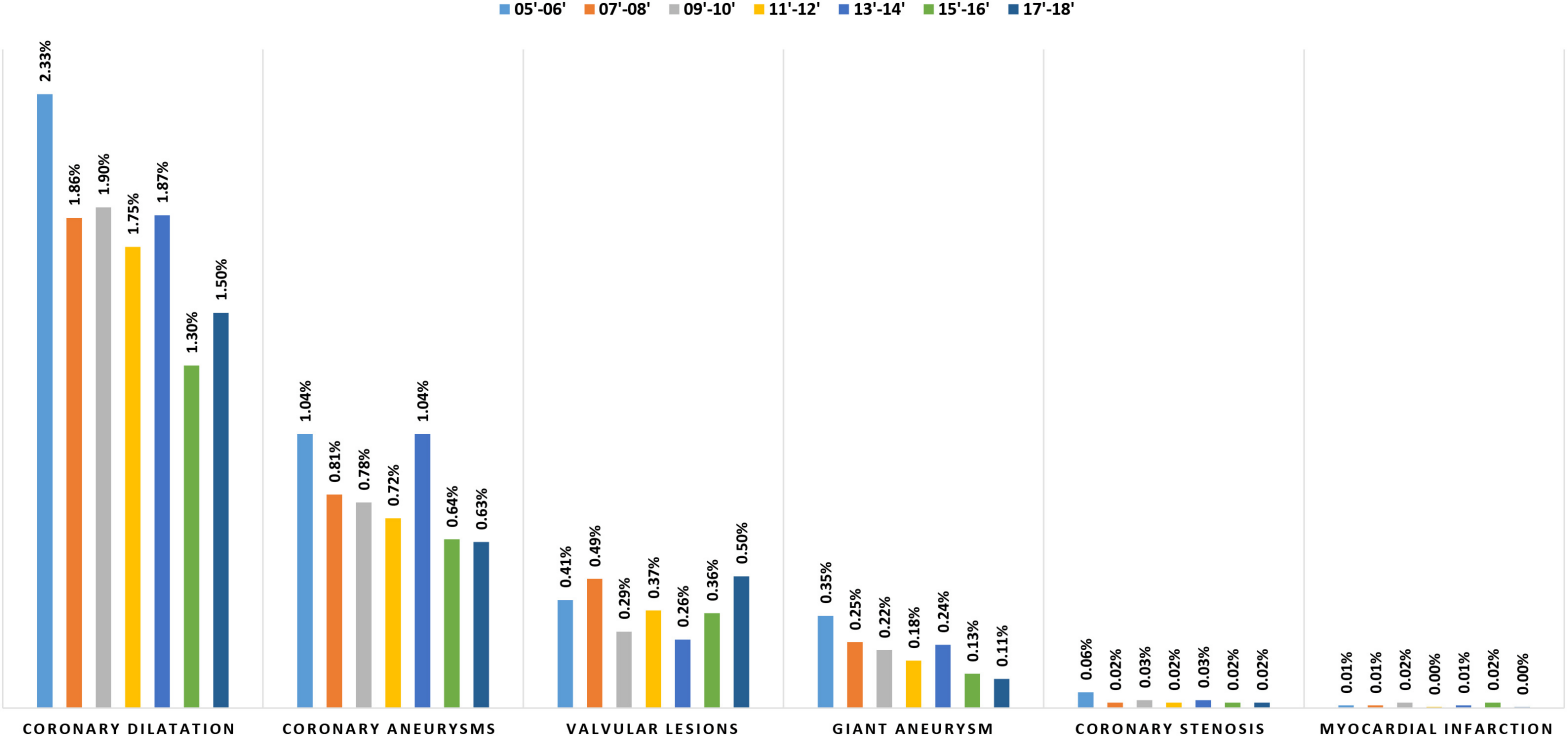
Annual frequency of cardiac sequelae [Source: Based on national survey from Japan (17)].

It has been reported from more than 60 countries after the first description in Japan (16). Based on a Japanese national (17), the number of KD patients has increased annually from 1995 to 2015. The frequency of atypical KD has increased from 9.8% in 1992 to 20.6% in 2016. The prevalence among 100,000 children aged 0–4 years was 319.6 in Japan, 170.9–194.9 in Korea, 71.9–110.0 in China, 49.4 in Hawaii, and 18.1–21.3 in the USA (18). The incidence was highest among Asian children, especially of Japanese ancestry. A 2006 USA survey (19) comprising 5,523 KD patients found that the incidence was higher in boys vs. girls (24.2 per 100,000 vs. 16.8 per 100,000, respectively). With the SARS-CoV-2 coronavirus pandemic, an increased number of children presenting with a novel syndrome sharing the features of KD and toxic shock syndrome, now named Multisystem inflammatory syndrome in children (MIS-C). Common overlapping clinical symptoms of MIS-C and KD include conjunctivitis, rash, red eyes, swollen hands and feet, red/cracked lips, swollen glands. Children with MIS-C had a broader age range, a deranged coagulation profile, significant gastrointestinal and neurologic symptoms. The laboratory findings also revealed significant elevation of cardiac and inflammatory markers, including Troponin, pro-B-type natriuretic peptide (proBNP), C-reactive protein (CRP), and erythrocyte sediment rate (ESR). Epidemiologic studies of MIS-C have suggested that younger children are more likely to present with KD-like features while older children are prone to develop myocarditis or myocardial dysfunction (20–26). Cardiovascular complications in the acute phase of KD were reported in 9.2% (10.2% of boys and 7.7% of girls) of patients in Japan's 25th national survey (27). Cardiovascular complications included coronary dilatation (6.52%), valvular lesions (1.55%), coronary aneurysms (0.95%), giant coronary aneurysms (0.11%), coronary stenosis (0.02%), and acute myocardial infarction (AMI) (0.01%). Cardiovascular sequelae reported in 2.76% (3.17% of boys and 2.26% of girls) of patients, included coronary dilatation (1.5%), coronary aneurysms (0.63%), valvular lesions (0.5%), giant coronary aneurysms (0.11%), coronary stenosis (0.02%), and myocardial infarction (MI) (0.003%). Except for valvular lesions, these sequelae occurred more often in boys. In addition, the incidence of coronary aneurysms and coronary dilatation was higher in patients with atypical KD. The frequency of cardiac sequelae from the annual survey in Japan is shown in Figure 1 (17).

## The Pathology of KD

### Causes and Pathogenesis

**Figure 2:**
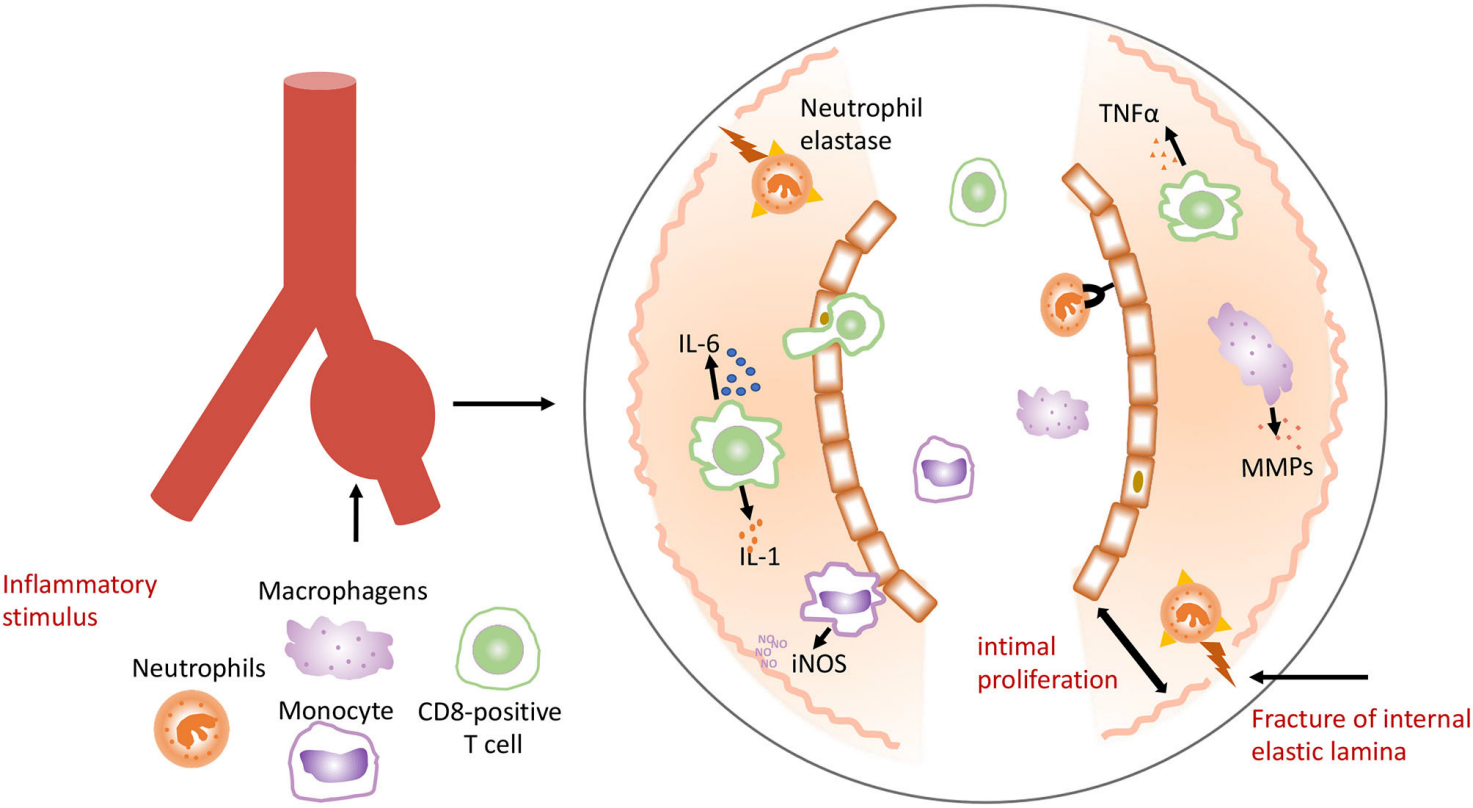
Inflammatory stimulus in Kawasaki syndrome.

The cause of KD remains unknown. It is speculated that this disease may be triggered by a novel RNA virus that enters through the upper respiratory tract, which leads to pathogen-induced immune response (28, 29). Researchers had found intracytoplasmic inclusion bodies of RNA virus in multiple cells throughout the KD patients' body. However, the molecular details of these inclusion bodies remain unknown. Epidemiological studies suggested that KD might be caused by conventional pathogens ranging from gram positive or atypical microorganisms (30). Activation of the immune system with high numbers of activated neutrophils, interleukin (IL)-1, IL-6, and tumor necrosis factor (TNF) was found in KD patients (31). Activated monocytes/macrophages seem to play an essential role in KD (31–33), which stimulate inflammatory cells to infiltrate and accumulate in the intima, and release proinflammatory molecules including IL-1, IL-6, TNFα, and matrix metalloproteinases (MMPs). Neutrophils elastase is released, which damages the internal elastic lamina and causes thickening. Macrophages secrete the inducible nitric oxide synthase (iNOS). This cascade of events leads to intimal proliferation, internal elastic destruction, and aneurysmal dilatation of the vessel (Figure 2) (34).

### Formation and Progress of Coronary Artery Aneurysms

**Figure 3:**
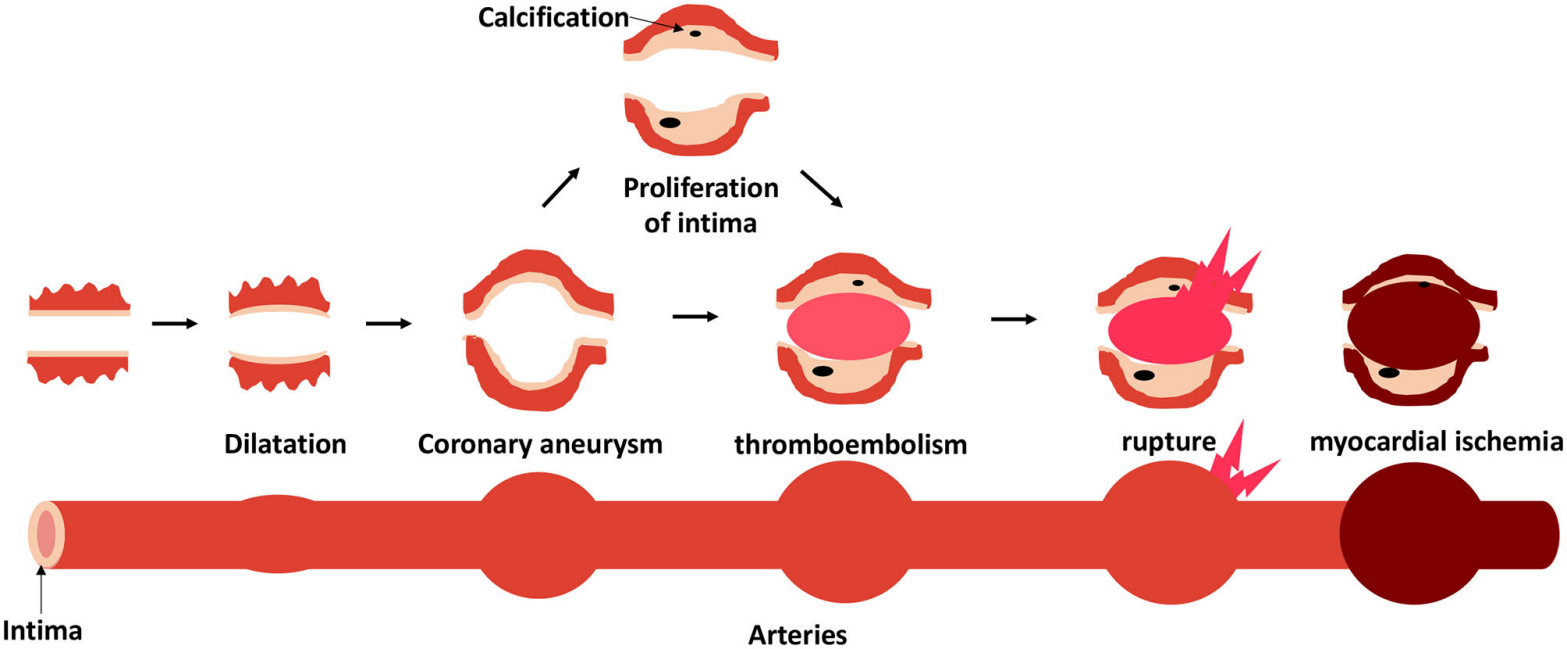
Long-term prognosis of coronary aneurysm.

In the early stages of KD, the synchronous neutrophilic process involving necrotizing arteritis is complete within 2 weeks after fever onset. It progressively destroys the arterial wall into the adventitia, causing the coronary artery lesions (CALs) to dilate. The internal and external elasticity become fragmented, causing aortic blood pressure to become unbearably high, and an aneurysm begins to form (35). If the internal diameter of an aneurysm increases excessively, rupture, or thromboembolism may occur. After the acute phase of KD, chronic vasculitis characterized by an asynchronous infiltration of plasma cells, lymphocytes, and eosinophils with decreased macrophages can continue for months or years in a small proportion of patients. At this phase, progressive thickening of the intima may lead to lumen stenosis. If clinical manifestations are evident, it can cause peripheral myocardial ischemia (Figure 3). Pathological outcomes of coronary artery aneurysms depend on the severity of the lesions. Slightly dilated arteries may have a chance to return to normal. Large or giant aneurysms have thickening intima and lost their elastica, which cannot be regenerated. Aneurysms and atherosclerotic lesions may occur in coronary artery branches (36).

### Potential Mechanisms of Atherosclerosis Formation

Carotid intima-media thickness (cIMT) (37, 38), abnormal lipid profile (39–41), including total cholesterol (TC), triglycerides (TG), low-density lipoprotein (LDL) cholesterol (LDL-C), arterial stiffness (42, 43), flow-mediated dilatation (FMD) (5) and inflammatory biomarkers [hsCRP (44) and, myeloperoxidase (45)] have been reported to be the risk factors for atherosclerosis. On the other hand, those markers mentioned earlier also appear in patients with KD history (5, 6, 11, 46). Potential mechanisms of increased risk for accelerated atherosclerosis include (1) arterial damage secondary to the acute process of the disease itself that may change the vascular structure and function, and propagate proatherogenic progress, (2) enduring inflammation and vasculitis that promote the development of atherosclerosis, and (3) patients with a history of KD may have other types of risk factors for atherosclerosis (47).

## Clinical Point of View: No Consensus

### Carotid Intima-Media Thickness

The cIMT is a noninvasive marker of atherosclerosis (38), and is considered a useful marker in adults (48). Recent research has focused on cIMT to determine if patients with KD history may be at risk of premature atherosclerosis (Table 1) (6, 43, 49–57). A few studies showed that the mean cIMT was significantly higher in KD patients than controls (*p* < 0.001) (6, 49, 52, 56, 57), while other studies did not show similar results (43, 50, 51, 53–55). Noto et al. (56) found significant differences between cases and controls, and in patients with KD history, atherosclerosis seemed to be age-dependent. The mean age of KD patients was 20.5. However, 26 out of the 35 patients included had persistent CAAs, and only 52% had received intravenous immunoglobulin (IVIG) during the acute episode. Gopalan et al. (49) found that the mean cIMT remained higher in patients with KD than those without KD at an average duration of 6.9 years after the acute episode. The authors suggested that children with KD may continue to have increased cIMT even several years after the acute phase. Watanabe et al. (58) found similar results. Virtual histological-intravascular ultrasonography findings were compared between patients with KD for <1 year (group A) and those with KD for >10 years (group B). There was no difference in the area percentage of atherosclerosis between the groups. However, the authors concluded that atherosclerotic-like findings exist in CAL in patients with KD, even within a year of onset. Investigators (6) found intima-media thickening in patients with or without CAL and detected long-term functional abnormalities in KD patients with regressed CAAs or angiographically normal coronary arterial. Several studies (51, 53, 55) did not find significant difference in cIMT between the patients with KD and controls given variations in the study population, consisting of a younger or older population or a small group of patients with giant aneurysms. The 2017 American AHA guidelines (15) and the 2020 Japanese JCS guidelines (18) used the coronary artery *Z-*scores to classify Kawasaki patients into five risk levels. The higher the risk level, the higher the likelihood of a cardiovascular event in the patient. The above-mentioned studies only described the presence of CAAs in patients but did not stratify them based on risk categories. This may be one of the reasons for various results between the studies. Including more high-risk patients with persistent CAAs would be more desirable to study the association of cIMT on disease severity.

**Table 1:** Studies on carotid intima-media thickness (cIMT) in patients with a history of KD.

**Author, year**	**Country**	***N***	**Age**	**Male (%)**	**Follow-up (years)**	**Persistent CAAs**	**cIMT (mm)**	***P***	**Treat (%)**	**Reference**
Gopalan, 2018	India	27	13.85 ± 2.75	74	6.97 ± 1.18	0	Cases: 0.54 ± 0.087	<0.001	100	(49)
							Control: 0.42 ± 0.036			
Parihar, 2017	India	20	11.5 ± 3.70	60	4.48 ± 1.88	0	Cases: 0.036 ± 0.015	0.791	100	(43)
							Control: 0.035 ± 0.076			
Chen, 2017	Australia	60	14.3	58	11.46 ± 5.60	15	Cases: 0.49 ± 0.05	0.80	92	(50)
							Control: 0.48 ± 0.06			
Ishikawa, 2013	Japan	24	7.9 ± 2.8	58	6.5 ± 1.7	4	Cases: 0.433 ± 0.029	0.906	100	(51)
							Control: 0.433 ± 0.028			
Meena, 2013	India	27	8.22 ± 2.6	74	2.45 ± 1.19	1	Cases: 0.500 ± 0.071	0.000	100	(52)
							Control: 0.417 ± 0.065			
Selamet Tierney, 2013	America	203	16.73 ± 4.21	60	11.6 (1.2–26)^*^	10	Cases: 0.45 ± 0.03	0.385	93	(53)
							Control: 0.43 ± 0.04			
Gupta-Malhotra, 2009	America	28	20.9 ± 6.0	68	16 ± 6	0	Cases: 0.49 ± 0.07	0.905	36	(54)
							Control: 0.48 ± 0.06			
Lee, 2009	Korea	25	12.6 ± 2.0	NM	>8	NM	Cases: 0.41 ± 0.19	>0.05	100	(55)
							Control: 0.50 ± 0.01			
Noto, 2009	Japan	35	20.5 ± 9.3	80	18.6 ± 8.4	26	Cases: 0.57 ± 0.15	<0.001	52	(56)
							Control: 0.46 ± 0.05			
Cheung, 2007	China	50	8.6 ± 2.8	66	6.6 ± 3.1	13	Cases: 0.41 ± 0.04	<0.001	90	(6)
							Control: 0.36 ± 0.04			
Dalla Pozza, 2007	Germany	20	12.1 ± 4.7	60	4.1 ± 3.6	NM	Cases: 0.45 ± 0.02	<0.001	100	(57)
							Control: 0.42 ± 0.01			

cIMT, carotid intima-media thickness; N, KD patients enrolled; NM, not mentioned; Treat (%), The percentage of KD patients involved that were treated with intravenous immunoglobulin (IVIG) infusion ± aspirin at the time of diagnosis;

* *median (range)*.

### Flow Mediated Dilatation

Vascular function test FMD can be measured and used as a noninvasive index of endothelial function (43). It uses ultrasound imaging to measure the arterial response during reactive hyperemia. Because of its noninvasive nature, FMD has recently been studied in KD patients (Table 2) (4, 5, 43, 51, 56, 59, 60). Most of the studies achieve statistically significant results (4, 5, 51, 56, 59). The percent FMD (%FMD) was significantly reduced in the KD group than the control group. Two studies (51, 59) categorized the participants into three groups for comparisons, group 1 with CAA/CAL, group 2 without CAA/CAL, and group 3 comprised healthy controls. They found the %FMD differed significantly among the three groups. KD patients, especially those with CAA /CAL, would be more likely to have severe endothelial damage. Two studies (43, 60) found no significant differences between cases and controls. However, compared to others, they had few or no persistent CAAs in KD groups. The long-term complications remain low for patients without coronary artery involvement (15), similar to those without KD diagnosis (61).

**Table 2:** Studies on flow-mediated dilatation (FMD) in patients with a history of KD.

**Author, year**	**Country**	***N***	**Age**	**Male (%)**	**Follow-up (years)**	**Persistent CAAs**	**%FMD**	***P***	**Treat (%)**	**Reference**
Parihar, 2017	India	20	11.48 ± 3.5	60	4.48 ± 1.88	0	Cases: 13.31 ± 10.41	0.874	100	(43)
							Control: 12.86 ± 7.09			
Ishikawa, 2013	Japan	24	7.9 ± 2.8	58	6.5 ± 1.7	4	Cases 1: 4.4	<0.05	100	(51)
							Cases 2: 9.1			
							Control: 13.9			
Ghelani, 2009	India	20	8.4 ± 2.3	65	2.1 ± 1.7	0	Cases: 5.7 ± 9.2	0.017	100	(4)
							Control: 12.2 ± 8.9			
Noto, 2009	Japan	35	20.5 ± 9.3	80	18.6 ± 8.4	26	Cases: 9.1 ± 2.7	<0.001	52	(56)
							Control: 13.3 ± 4.8			
Liu, 2009	China	41	7.15 (3–11)^*^	61	4.4 (1.5–10)^*^	21	Cases 1: 4.5 ± 1.5	<0.01	100	(59)
							Cases 2: 9.5 ± 2.8			
							Control: 12.1 ± 2.3			
Niboshi, 2008	Japan	35	27.0 ± 4.2	46	24.1 ± 4.5	9	Cases: 10.4 ± 2.6	<0.01	NM	(5)
							Control: 14.4 ± 3.2			
Borzutzky, 2008	Chlie	11	10.6 ± 2.0	64	8.1 ± 3.6	1	Cases: 11.1 ± 5.7	NS	100	(60)
							Control: 8.0 ± 2.9			

Cases 1, KD with CAA /CAL; Cases 2, KD without CAA/CAL; FMD, flow-mediated dilatation; N, KD patients enrolled; NM, not mentioned; NS, not statistically significant (statistical significance was assumed at P < 0.05); Treat (%), The percentage of KD patients involved that were treated with intravenous immunoglobulin (IVIG) infusion ± aspirin at the time of diagnosis; %FMD: the percent FMD;

* *median (range)*.

### Lipid Profile

Determination of lipid profile is a part of risk stratification for atherosclerosis. The protective role of high-density lipoprotein (HDL) and the pathogenic role of LDL, TC, and TG in atherosclerosis are well-established. Some investigators (Table 3) studied the lipid profile between KD patients and controls to determine the differences (5, 47, 50, 54, 56, 57, 60, 62, 63). A majority of the existing studies indicated no differences between KD patients and controls on the lipid profile. However, one study (63) had a statistically significant result. The authors found that KD patients had significantly lower TC (*P* < 0.001), LDL (*P* < 0.001), and TG (*P* = 0.008) than those controls. Unlike other studies, the authors used nuclear magnetic resonance (NMR) spectroscopy to directly quantify the number of LDL and HDL particles and their size distribution because of its accurate assessment of atherosclerotic risk. The authors recommended managing KD patients with documented hyperlipidemia more proactively.

**Table 3:** Studies on lipid profile in patients with a history of KD.

**Author, year**	**Country**	**Age**	**Male (%)**	**LP (mg/dl)**	**Patients with KD, *n***	**Healthy controls, n**	***P***	**Reference**
Chen, 2017	Australia	14.3	58	TC	159.06 ± 33.67 (60)	169.51 ± 39.86 (60)	NS	(50)
				LDL-C	89.01 ± 29.41 (60)	96.75 ± 27.09 (60)	NS	
				HDL-C	54.95 ± 13.93 (60)	58.05 ± 13.16 (60)	NS	
				TG	70.88 (60)	70.88 (60)	NS	
Laurito, 2014	Italy	10 ± 3.7	64	TC	167 ± 33 (14)	157 ± 29 (14)	0.40	(62)
				LDL-C	91 ± 23 (14)	84 ± 21 (14)	0.37	
				HDL-C	60 ± 15 (14)	55 ± 14 (14)	0.39	
				TG	82 ± 38 (14)	89 ± 79 (14)	0.78	
Lin, 2014	USA	5.4	65	TC	148 (192)	169 (45)	<0.001	(63)
				LDL-C	85 (192)	106 (45)	<0.001	
				HDL-C	50 (192)	48 (45)	0.13	
				TG	82 (192)	105 (45)	0.008	
Gupta-Malhotra, 2009	USA	20.9 ± 6.0	68	TC	175 ± 36 (28)	157 ± 33 (27)	0.034	(54)
				LDL-C	103 ± 30 (28)	90 ± 23 (27)	0.076	
				HDL-C	52 ± 14 (28)	50 ± 13 (27)	0.180	
				TG	99 ± 48 (28)	86 ± 54 (27)	0.127	
Noto, 2009	Japan	20.5 ± 9.3	80	TC	172.8 ± 34.5 (35)	165.0 ± 21.2 (35)	0.43	(56)
				LDL-C	94.4 ± 23.8 (35)	90.2 ± 17.3 (35)	0.56	
				HDL-C	60.3 ± 12.1 (35)	56.4 ± 16.8 (35)	0.44	
				TG	91.0 ± 46.1 (35)	83.8 ± 42.6 (35)	0.63	
Niboshi, 2008	Japan	27.0 ± 4.2	46	TC	168.3 ± 27.9 (35)	161.3 ± 24.5 (36)	0.242	(5)
				LDL-C	97.3 ± 25.3 (35)	93.2 ± 19.4 (36)	0.454	
				HDL-C	56.5 ± 12.8 (35)	55.4 ± 8.9 (36)	0.690	
				TG	–	–	–	
Borzutzky, 2008	Chile	10.6 ± 2.0	64	TC	152.6 ± 27.9 (11)	150.5 ± 27.4 (11)	NS	(60)
				LDL-C	77.4 ± 20.8 (11)	83.6 ± 21.1 (11)	NS	
				HDL-C	58.6 ± 10.6 (11)	50.8 ± 10.8 (11)	NS	
				TG	83.2 ± 37.8 (11)	80.4 ± 31.5 (11)	NS	
McCrindle, 2007	Canada	15.5 ± 2.3	67	TC	160.99 ± 23.99 (52)	157.89 ± 27.09 (60)	0.52	(47)
				LDL-C	97.52 ± 21.67 (52)	94.04 ± 22.06 (60)	0.43	
				HDL-C	44.12 ± 10.06 (52)	46.05 ± 11.99 (60)	0.40	
				TG	97.46 ± 37.21 (52)	88.60 ± 36.33 (60)	0.22	
Dalla Pozza, 2007	Germany	12.1 ± 4.7	60	TC	169.4 ± 16.7 (20)	167.3 ± 18.4 (28)	NS	(57)
				LDL-C	94.3 ± 22.4 (20)	92.5 ± 16.4 (28)	NS	
				HDL-C	48.5 ± 11.2 (20)	47.7 ± 17.9 (28)	NS	
				TG	123.6 ± 55.6 (20)	130.5 ± 65.3 (28)	NS	

*HDL-C, High-density lipoprotein cholesterol; LDL-C, low-density lipoprotein cholesterol; LP, lipid parameter; NS, not statistically significant (Statistical significance was assumed at P < 0.05); TC, total cholesterol; TG, triglycerides*.

### High-Sensitivity C-Reactive Protein or C-Reactive Protein

Some studies support the role of the inflammatory mechanisms in atherogenesis (44, 64, 65). Leukocyte recruitment and proinflammatory cytokines are crucially in the early phase of atherogenesis (44). Serum hsCRP, an indicator of inflammation, is a reliable clinical marker to predict the risk of coronary events (11). Several studies (Table 4) discussed (hs) CRP in follow-up patients of KD recent years (5, 42, 50, 51, 54, 57, 60). The inflammatory process was more severe in KD patients with persistent CAL than those without, manifested with prolonged duration of fever and increasing CRP levels (51). The authors hypothesized the longer the duration of fever, the greater the risk of inflammation-induced endothelial dysfunction in KD patients. Another study showed similar result (57). Borzutzky et al. (60) found that patients with elevated hsCRP did not have persistent CAL, suggesting that inflammation may also be present in the large subgroup of children without persistent CAL. Niboshi et al. (5) found no statistically significant difference between cases and controls. The investigators classified the KD patients into three groups: group A with CAL; group B with transient CAL; and group C without CAL. The results revealed a significant increase in group A as compared to the control group. In contrast with aforementioned studies, several other studies (42, 50, 54) had negative results. Cho et al. (42) revealed that levels of hsCRP were not significantly different between KD and healthy subjects, however, homocysteine level, another indicator of inflammation, was significantly higher in KD groups. And one of the studies that had negative results (54), compared to others, had no persistent CAAs in KD groups. Therefore, CPR/hsCRP should not be used as the only inflammatory marker when examining patients with KD. The combined use of several biomarkers would help predict association with disease severity and development of CAAs.

**Table 4:** Studies on C-reactive protein (CRP)/high-sensitive C-reactive protein (hsCRP) in patients with a history of KD.

**Author, year**	**Country**	***N***	**Age**	**Male (%)**	**Follow-up (year)**	**Persistent CAAs**	**(hs)CRP (mg/dl)**	***P***	**Treat (%)**	**Reference**
Chen, 2017	Australia	60	14.3	58	11.46 ± 5.60	15	Cases: 0.06^*^	NS	100	(50)
							Control: 0.04^*^			
Cho, 2014	Korean	68	7.61 ± 1.69	59	5.05 ± 2.43	8	Cases 1: 0.91 ± 0.72^*^	NS	91	(42)
							Cases 2: 1.32 ± 1.69^*^			
							Control: 1.17 ± 0.54^*^			
Ishikawa, 2013	Japan	24	7.9 ± 2.8	58	6.5 ± 1.7	4	Cases 1: 15.4^#^	0.022	100	(51)
							Cases 2: 7.0^#^			
Gupta-Malhotra, 2009	USA	28	20.9 ± 6.0	68	16 ± 6	0	Cases: 0.24^*^	0.118	36	(54)
							Control: 0.20^*^			
Niboshi, 2008	Japan	35	27.0 ± 4.2	46	24.1 ± 4.5	9	Cases 0: 0.153 ± 0.32^*^	<0.05	NM	(5)
							Control: 0.035 ±0.05^*^			
Borzutzky, 2008	Chile	11	10.6 ± 2.0	64	8.1 ± 3.6	1	Cases: 0.23 ± 0.3^*^	0.045	100	(60)
							Control: 0.05 ± 0.03^*^			
Dalla Pozza, 2007	Germany	20	12.1 ± 4.7	60	4.1 ± 3.6	NM	Cases 1: 14.0 ± 6.2^#^	<0.05	100	(57)
							Cases 2: 3.0 ± 3.9^#^			

*Cases 0, KD with persistent CAA/CAL; Cases 1, KD with CAA/CAL; Cases 2, KD without CAA/CAL; CRP, C-reactive protein; hsCRP, high-sensitive C-reactive protein; N: KD patients enrolled; NM, not mentioned; NS, not statistically significant (statistical significance was assumed at P < 0.05); Treat (%), The percentage of KD patients involved that were treated with intravenous immunoglobulin (IVIG) infusion ± aspirin at the time of diagnosis*.

* The value of hsCRP;

# *The value of CRP*.

## Long-Term Management

### Evaluation and Lifestyle Change

It remains unknown if atherosclerotic risk factors affect the long-term progression of KD (15). Nonetheless, KD patients have been classified as a risk condition of atherosclerotic cardiovascular disease (CVD) and targeted in the evaluation of atherosclerotic CVD. KD patients with current coronary aneurysms are considered high risk, and those with regressed aneurysms are at moderate risk (66). Other risk factors include family history, age, gender, diet, physical inactivity, tobacco exposure, blood pressure, lipid levels, obesity, diabetes mellitus, predisposing conditions, metabolic syndrome, inflammatory markers and perinatal factors (66). Although it is challenging to recognize the progression of atherosclerosis in patients with KD history, coronary endothelial dysfunction seems to be present in those with CAL. A recent scientific statement from the Japanese Circulation Society (JCS) (18) recommends eliminating arteriosclerosis-promoting factors in patients with CAL, and lifestyle modifications including smoking cessation, prevention of obesity, and healthy diet, are essential for patients with KD history.

### Medical Treatment of CAL

#### Statins

Statins have been the cornerstone of therapy for preventing atherosclerotic cardiovascular events in adults (67). Based on the American College of Cardiology (ACC)/AHA Guidelines for the primary prevention of cardiovascular disease, statin therapy is the first-line agent for primary prevention in patients at an increased risk for atherosclerotic events. Statins have beneficial effects on inflammation, endothelial function, oxidative stress (68–71). Although controversy remains on whether pathology of KD may have features of atherosclerosis, statins have been recently recommended for empirical therapy in KD patients with past or current aneurysms (15, 18) according to the AHA and JCS. Some studies (10, 72) reported that KD patients with aneurysms had shown statistically significant improvement in reductions in hsCRP and improved endothelial function after 3 months of statin therapy. In another small study, short-term statin therapy for 3 months seemed to significantly improve chronic vascular inflammation and endothelial dysfunction in children with KD with little to no adverse effects (73).

#### Angiotensin II Receptor Blocker, Angiotensin Converting Enzyme Inhibitor

Vascular stenosis is formed by intimal proliferation in KD patients, which is caused by the action of the renin-angiotensin system (RAS) in the vascular wall (18). Investigators found that angiotensin II (Ang II) has significant proinflammatory actions in the vascular wall, inducing inflammatory cytokines and adhesion molecules (74). It is hypothesized that RAS antagonists (ARB, ACEI) can prevent atherosclerosis by reducing vascular inflammation (75). They have also been shown to be protective against atherosclerotic CVD (76). JCS recommends ARB and ACEI for preventing coronary artery stenosis in KD patients with CAL (18).

#### β-Blockers

β-blockers have an essential role in the management of the atherosclerotic disease (77, 78) by inhibiting the sympathetic system, exhibiting antioxidant and anti-inflammation effects (79). Their roles have also been extended to the KD coronary disease. AHA 2017 guidelines recommend β-blockers be considered for KD patients with persistent large or giant aneurysms because they are at high risk of developing MI (15).

## Summary

Whether the long-term pathological vascular processes in patients with KD history are distinct vasculopathy or typical atherosclerosis features remains controversial. In this article, we reviewed the pathology of KD and its possible role in atherosclerosis development. The risk factors for atherosclerosis have also existed in patients with KD history. Upon reviewing the recent data regarding whether KD history predisposes patients to premature atherosclerosis, there was no conclusive consensus. However, timely diagnosis and treatment of KD is critical to prevent cardiovascular complications. In addition, consistent follow-up visits may be necessary. Counseling on lifestyle factors affecting atherosclerosis, including dyslipidemia, hypertension, smoking, and obesity, is an essential aspect of long-term management in all patients with a history of KD. Moreover, it may be wise to give pharmacotherapy empirically for KD patients with past or present aneurysms.

## Author Contributions

FC and Y-YZ: conceptualization. Y-YZ and SK: writing—original draft preparation. FC and MZ: writing—review and editing. FC: funding acquisition. All authors have read and agreed to the published version of the manuscript.

## Conflict of Interest

The authors declare that the research was conducted in the absence of any commercial or financial relationships that could be construed as a potential conflict of interest.

## Abbreviations

ACC: American College of Cardiology
ACEI: angiotensin II receptor blocker
ACS: acute coronary syndrome
AHA: American Heart Association
AMI: acute myocardial infarction
Ang II: angiotensin II
ARB: angiotensin converting enzyme inhibitor
CAAs: coronary artery abnormalities
CALs: coronary artery lesions
cIMT: Carotid intima-media thickness
CRP: C-reactive protein
CVD: cardiovascular disease
FMD: flow-mediated dilatation
hsCRP: high-sensitivity C-reactive protein
HDL: high-density lipoprotein
HDL-C: high-density lipoprotein cholesterol
IL: interleukin
IVIG: intravenous immunoglobulin
JCS: Japanese Circulation Society
KD: Kawasaki disease
LDL: low-density lipoprotein
LDL-C: low-density lipoprotein cholesterol
MI: myocardial infarction
MMPs: matrix metalloproteinases
NMR: nuclear magnetic resonance
RAS: renin-angiotensin system
TC: cholesterol
TG: triglycerides
TNF: tumor necrosis factor
%FMD: the percent flow-mediated dilatation.
